# Assessing Nutraceuticals for Hepatic Steatosis: A Standardized In Vitro Approach

**DOI:** 10.3390/nu18030388

**Published:** 2026-01-24

**Authors:** Victoria E. J. M. Palasantzas, Dicky Struik, Trijnie Bos, Sebo Withoff, Jingyuan Fu, Johan W. Jonker, Joanne A. Hoogerland

**Affiliations:** 1 Department of Pediatrics, University Medical Center Groningen, 9713 GZ Groningen, The Netherlands; v.e.j.palasantzas@umcg.nl (V.E.J.M.P.);; 2 Department of Genetics, University Medical Center Groningen, 9700 RB Groningen, The Netherlands

**Keywords:** MASLD, nutraceuticals, steatosis, intracellular triglycerides, HepG2, Fa2N-4, short-chain fatty acids, antioxidants, polyphenols, resmetirom

## Abstract

**Background/Objectives:** Nutraceuticals, including short-chain fatty acids (SCFAs) and antioxidants (AOXs), are nutrient-derived bioactive compounds considered as potential treatments for metabolic-associated steatotic liver disease (MASLD). However, in vitro studies of their effects are limited by inconsistent experimental conditions, including differences in cell lines, methods of steatosis induction, and culture media, and by reliance on qualitative rather than quantitative assessments. Here, we systematically evaluate the anti-steatotic potential of eight commonly used nutraceuticals—three SCFAs (butyrate, acetate, and propionate) and five AOXs (resveratrol, curcumin, berberine, chlorogenic acid, and vitamin E)—using a standardized in vitro approach. **Methods:** Following a systematic literature review to identify common experimental conditions, we developed an assay to validate steatosis induction and quantified the effects of the nutraceuticals. For our studies we used the HepG2 liver cancer cell line and the Fa2N-4 immortalized hepatocyte cell line. Steatosis was modeled by stimulating cells with free fatty acids and fructose for 48 h. Nutraceuticals were added either concurrently with steatotic stimulation, to assess preventive effects, or after 24 h to assess therapeutic effects. Anti-steatotic drugs (resmetirom, semaglutide, obeticholic acid, and a DGAT2 inhibitor) were included as positive controls. Intracellular triglyceride levels were measured to quantify steatosis. **Results:** A systematic review of 46 studies revealed large differences in culture conditions, steatosis induction, and nutraceutical assessment. In our experiments, most nutraceuticals did not reduce intracellular triglycerides, with the exception of vitamin E. Surprisingly, butyrate, berberine, and curcumin increased triglyceride accumulation. Resmetirom was the only drug that significantly decreased triglycerides, while obeticholic acid, semaglutide, and the DGAT2 inhibitor showed minimal or inconsistent effects. Fa2N-4 cells were generally more sensitive than HepG2 cells, showing larger absolute changes in triglyceride levels in response to both nutraceuticals and resmetirom. **Conclusions:** We established a standardized in vitro assay to evaluate the anti-steatotic potential of nutraceuticals. Using this system, we found that SCFAs and AOXs did not consistently reduce intracellular triglycerides, highlighting the need for quantitative assessments and careful validation when studying anti-steatotic interventions in vitro.

## 1. Introduction

Metabolic dysfunction-associated steatotic liver disease (MASLD) is the most prevalent chronic liver disease, affecting nearly 40% of the global population [1]. MASLD encompasses a spectrum of liver conditions, ranging from simple hepatic steatosis (fat accumulation) to metabolic-associated steatohepatitis (MASH), which can progress to fibrosis, cirrhosis, hepatocellular carcinoma, and end-stage liver disease. Extrahepatic features include insulin resistance, abdominal obesity, atherogenic dyslipidemia, and hypertension [2]. The pathogenesis of MASLD involves multiple metabolic disturbances, including excessive lipid influx from high-calorie diets, insulin resistance-driven adipose lipolysis, and increased de novo lipogenesis. Impaired fatty acid oxidation and export, mitochondrial dysfunction, and endoplasmic reticulum stress further exacerbate hepatic steatosis [3]. Lifestyle factors such as diet and physical activity influence disease onset, while genetic predisposition and gut microbiota dysbiosis modulate disease severity [4,5,6].

Although pharmacological interventions have recently emerged, US FDA-approved therapies remain limited. Resmetirom received conditional approval for treatment of MASH in 2024, followed by semaglutide in 2025 [7,8,9]. However, the response rates are modest (29.9% for resmetirom, 59% for semaglutide) [10,11]. Therefore, dietary interventions focusing on reducing caloric intake from high-fat and high-carbohydrate sources remain the first-line treatment [6]. In addition, functional dietary components, or ‘nutraceuticals,’ may offer targeted strategies for MASLD and early MASH. Nutraceuticals are bioactive molecules derived from food and include nutrients, herbal compounds, and phytochemicals [12,13]. Common examples are short-chain fatty acids (SCFAs), vitamin E, and phenolic acids such as curcumin and chlorogenic acid.

SCFAs are fatty acids with fewer than six carbon atoms, including acetate (C2), propionate (C3), and butyrate (C4), produced by intestinal microbiota through dietary fiber fermentation. SCFAs have been shown to improve hepatic steatosis in in vitro and preclinical models. For instance, butyrate reduced triglyceride accumulation in an in vitro gut-liver chip model using HepG2 and Caco-2 cells [14], and SCFAs improved metabolic function, restored gut barrier integrity, and regulated satiety in rodent models [15,16]. Mechanistically, SCFAs modulate lipid metabolism via PPAR-dependent shifts from lipogenesis to fatty acid oxidation, influencing SREBP-1c, PPARα, and CPT1A-AMPKα1-ACC pathways, and they may also act through histone deacetylase (HDAC) inhibition [14,17,18,19]. Animal studies suggest that the liver is a critical target for the effect of butyrate, as butyrate’s anti-steatotic effect was absent in mice that lacked hepatic PPARγ [17]. Clinical trials, however, could not demonstrate a causal improvement in hepatic steatosis following SCFA supplementation [20,21].

Phytochemicals, including polyphenols such as resveratrol, curcumin, berberine, chlorogenic acid, and vitamin E, are recognized for their antioxidant properties. They can protect hepatocytes from oxidative stress induced by lipid accumulation and exhibit anti-steatotic and anti-inflammatory effects in animal models and cell lines [21,22,23]. Vitamin E is currently the only phytochemical recommended off-label for MASLD patients without diabetes [21,24], although the evidence for its efficacy in improving steatohepatitis and fibrosis is limited and hepatotoxicity has been reported [25,26,27].

The aforementioned nutraceuticals and their main functions as well as protective (molecular) mechanisms in the context of MASLD are summarized in Table 1.

Recently, pharmaceutical interventions such as obeticholic acid, resmetirom, and semaglutide have gained attention (Table 2). While obeticholic acid was rejected for the treatment of MASH due to uncertain histopathological benefits versus risks [66,67,68], resmetirom and semaglutide have been approved [8,9]. Additionally, emerging compounds targeting hepatic triglyceride synthesis, including DGAT2 inhibitors, are under preclinical investigation [69,70,71].

Despite extensive mechanistic studies (Table 1), robust evidence demonstrating that nutraceuticals can reduce or reverse hepatic steatosis in terms of lipid accumulation is lacking. A major barrier is the absence of a standardized in vitro model to test anti-steatotic effects. Many studies investigate the anti-steatotic potential of nutraceuticals, yet all studies use different lipid/fructose stimulation, differ in stimulation duration, treatment duration and timing, and they do not always assess steatosis with a quantitative endpoint, such as absolute triglyceride levels. There has not been a standardized approach defined to assess the quantitative anti-steatotic effects of various nutraceuticals.

In this study, we defined such a model through a systematic review of cell-based studies on nutraceuticals in steatosis. We then used this assay to assess the anti-steatotic properties of SCFAs (butyrate, acetate, and propionate) and polyphenolic antioxidants (resveratrol, berberine, curcumin, chlorogenic acid, and vitamin E). Using the most reported conditions from the literature, we quantitatively measured intracellular triglycerides in two hepatocyte cell lines: tumor-derived HepG2 cells and immortalized hepatocyte-derived Fa2N-4 cells. Based on our literature review, we aim to standardize anti-steatotic cell line-based screening, from steatotic induction to pharmacological intervention, to improve the reliability of in vitro therapeutic investigations for MASLD.

## 2. Materials and Methods

### 2.1. Chemicals

To assess the potential anti-steatotic potency of nutraceuticals, chemicals were obtained from Sigma Aldrich (Merck KGaA, Darmstadt, Germany). SCFAs used in this study include sodium butyrate (catalog #303410), sodium acetate (catalog #S8750), and sodium propionate (catalog #P1880). Sodium chloride (catalog #106.404) was used as the vehicle control. The following plant-derived nutraceuticals were used: resveratrol (catalog #554325), curcumin (catalog #C7727), berberine (catalog #B3412), chlorogenic acid (catalog #C3878), and (±)-α-Tocopherol (vitamin E) solution (catalog #V-020).

### 2.2. Cell Culture

To study intracellular hepatocyte effects, HepG2 cells were obtained from ATCC (catalog #HB-8065, Manassas, VA, USA) and cultured in Dulbecco’s Modified Eagle’s Medium (DMEM) with GlutaMAX (catalog #10569010, Thermo Fisher Scientific, Waltham, MA, USA) containing 25 mM of glucose and supplemented with 10% heat-inactivated fetal bovine serum (FBS) (catalog #A5256701, Gibco, Thermo Fisher Scientific) and 1% penicillin-streptomycin (catalog #15140122, Thermo Fisher Scientific). Fa2N-4 cells [79] were obtained via Tebubio (catalog #IFH15, Tebubio, Le Perray-en-Yvelines, France) and cultured on coated plates with 0.02 mg/mL rat tail collagen-I (catalog #354249, Corning, New York, NY, USA) in Williams E medium with GlutaMAX (catalog #32551087, Gibco, Thermo Fisher Scientific) containing 11 mM glucose and supplemented with 10% heat-inactivated FBS (catalog #A5256701, Gibco, Thermo Fisher Scientific), 1% penicillin-streptomycin (catalog #15140122, Thermo Fisher Scientific), 20 mU/mL insulin (100 U/mL injection vial, Sanofi, Paris, France) and 100 nM dexamethasone (20 mg/mL stock, catalog #9265331, Centrafarm, Breda, The Netherlands). For experiments, cells were seeded in 24-well plates at a density of 105,000 cells per cm^2^ and left overnight to attach. Both cell lines were confirmed to be authentic and not cross-contaminated with other cell lines using short-tandem repeat profiling (Eurofins, Luxembourg City, Luxembourg).

To mimic hepatic steatosis in vitro, cells with a confluency of 60–70% were stimulated with culture media supplemented with 1 mM fructose (D-(−)-Fructose, F0127, Sigma, Merck KGaA) and a mixture of free fatty acids (FFA), palmitic acid, and oleic acid (catalog #03880 and P9767, Sigma Aldrich, Merck KGaA) in a 1:2 ratio for 24 h or 48 h. A total of 10 mM palmitic acid or oleic acid was dissolved in 100% EtOH, dried under gaseous N_2_, and dissolved in 10% bovine serum albumin (BSA) (catalog #A6003, Sigma, Merck KGaA) (pH adjusted to 7.4). BSA was used as a vehicle control. Cells were stimulated with 600 µM FFAs for 24 h unless otherwise specified.

### 2.3. Nutraceuticals and Pharmaceuticals

To measure the influence of various compounds on intracellular triglyceride levels, steatotic cells were stimulated with nutraceuticals or pharmaceuticals. The treatment approach was either at the same time as steatotic stimuli for 48 h (‘co-stimulation’, prevention) or after 24 h of steatotic stimuli exposure (‘post-stimulation’, intervention) (Table 2). Concentrations of nutraceuticals were based on consensus in Appendix A and cellular toxicity, relevant human blood levels, and drug ADME (absorption, distribution, metabolism, and excretion), including bioavailability (Appendix B). For pharmaceuticals, we used literature references, indicated in Table 3 below, previously cited to have at least 60% cell viability in HepG2 cells.

### 2.4. Triglyceride Quantification

To quantify intracellular triglycerides, we collected cell lysates. Briefly, cells were washed twice with 1x phosphate-buffered saline (PBS) (catalog #14190-169, Thermo Scientific) and collected in 1x Tris-buffered saline (TBS; home-made) to quantify intracellular triglycerides. Cells were lysed by sonication for 10 s at 40% amplitude. Prior to fat isolation, protein levels were measured using the Bicinchoninic Acid kit (BCA^TM^ Protein Assay Kit, catalog #23227, Pierce, Thermo Fisher Scientific) [82], and the sample input for fat isolation was equalized to the sample with the lowest protein concentration.

Lipids were extracted according to the Bligh and Dyer lipid extraction [83]. In short, chloroform/methanol (2:1) was used to separate lipids by vortexing and centrifugation of the samples. Samples were dried under a N_2_ flow, dissolved in 2% Triton in chloroform, vortexed, and left to dry. Next, samples were dissolved in demi water and incubated for one hour at 37 °C. The concentration of triglycerides was measured by an enzymatic colorimetric Triglycerides FS (catalog #157109910917, Diasys Diagnostic Systems GmbH, Holzheim, Germany) assay and normalized to protein. Precimat glycerol (catalog #10166588, Roche Diagnostics GmbH, Mannheim, Germany) was used as a reference (0.82–105 µM/mL) to make a standard curve and interpolate our samples.

### 2.5. Literature Review

To assess the existing literature, we conducted a literature search to identify relevant studies on nutraceuticals and their anti-steatotic effects. The search was performed using electronic databases including PubMed and Google Scholar. Keywords used included combinations of: “[nutraceutical] AND “steatosis”, “[nutraceutical] AND cell culture”, and “[nutraceutical] AND in vitro”. The nutraceuticals searched were: “butyrate”, “acetate”, “propionate”, “resveratrol”, “curcumin”, “berberine”, “chlorogenic acid”, and “vitamin E”. No formal search protocol or PRISMA guidelines were followed, as this review was exploratory in nature. Our selection criteria included the use of human in vitro hepatocyte cell lines in the published data, and only original research papers were included.

We included 46 independent studies, including some that explored multiple nutraceuticals. Therefore, 55 entries are reported, with each compound listed separately. We extracted information on the cell lines used, the media, the screening methodology, and the readouts. As no quantitative meta-analysis was performed, this review is subject to bias and may not comprehensively cover all relevant studies. The aim was to provide a broad overview rather than an exhaustive analysis.

### 2.6. Statistical Analysis

Statistical analyses were performed using BrightStat (Version 1.3.1, ‘https://secure.brightstat.com/index.php’ (accessed on 23 July 2025) [84]). Differences between groups with identical vehicles were tested using the unpaired Mann–Whitney U test (control versus treated) or Kruskal–Wallis (for >2 conditions). All values are reported as the mean of ≥3 replicates. Values with *p* < 0.05 were considered significant. Data visualization was conducted using GraphPad Prism (Version 10.4.1 (532), San Diego, CA, USA).

## 3. Results

### 3.1. Systematic Review of Studies on Nutraceutical Effects in Hepatic Steatosis

**Figure 1:**
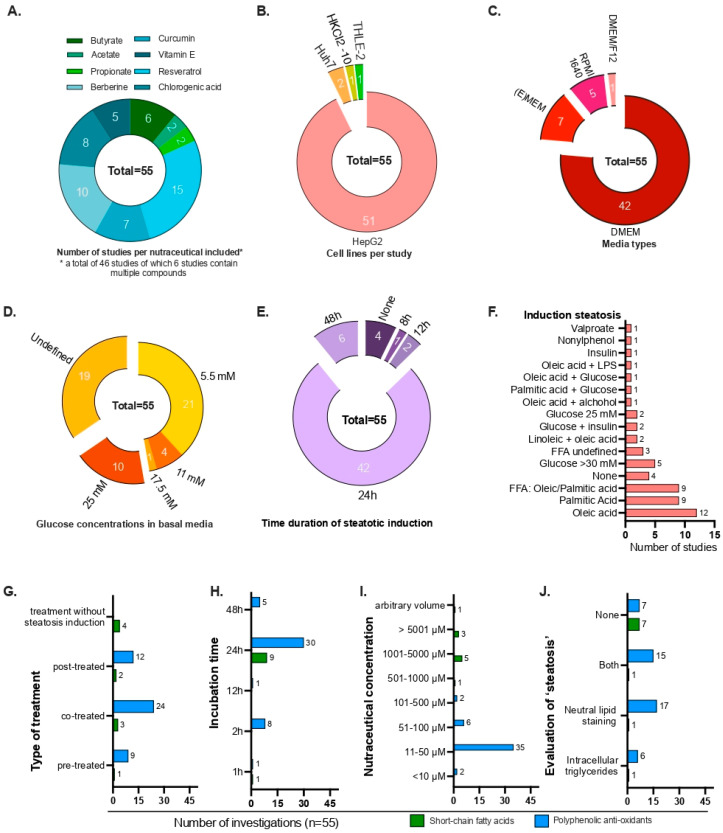
Literature overview on conditions under which nutraceuticals are investigated in the context of hepatic steatosis in human hepatocyte cell lines. (**A**) Number of studies per included nutraceutical, (**B**) cell lines used, (**C**) culture media used, (**D**) glucose concentration in culture media used, (**E**) duration of steatotic stimulation, (**F**) stimulations used to mimic steatotic-like phenotype, (**G**) type of treatment with nutraceuticals per study, (**H**) incubation time under which the effect of nutraceutical groups were studied, (**I**) nutraceutical concentrations primarily studied in included studies and (**J**) assessment of steatosis by neutral lipids.

To investigate the impact of nutraceuticals on hepatic steatosis, we conducted a systematic literature review and assessed the degree of consensus across studies. We analyzed 46 studies that reported on the anti-steatotic properties of nutraceuticals using human hepatocyte-derived cell lines (Appendix A). In total, we identified 55 experimental entries, as some studies evaluated multiple compounds. The studies were analyzed for variations in cell lines, culture conditions, steatosis induction, and nutraceutical treatment protocol (Figure 1A).

Most selected studies (51 out of 55) investigating nutraceutical effects employed two-dimensional hepatocyte cell lines, primarily the hepatoblastoma-derived HepG2 cell line (Figure 1B). The cancerous origin of the HepG2 cell line affects its metabolic capacity, as reflected by the downregulation of PPAR signaling and cytochrome P450 drug-metabolizing enzymes [85,86]. The choice of basal culture medium also varied considerably (Figure 1C). Among the reviewed studies, 76% (42 out of 55) used DMEM-based media with glucose concentrations ranging from 5.5 mM to 25 mM (Figure 1D). However, most studies did not specify the exact DMEM formulation used. Experimental induction of steatosis varied widely across studies, using glucose, insulin, FFA, or combinations thereof (Figure 1E,F). Some studies additionally included serum starvation or glucose reduction steps before stimulation (Appendix A). Disease induction periods were generally short, typically 24 h, despite MASLD being a chronic condition (Figure 1E). The timing and duration of nutraceutical exposure also differed markedly, complicating comparisons between studies. Many studies applied nutraceuticals either concurrently with steatotic stimuli (“prevention” or “co-treated”) or after induction (“intervention” or “post-treated”) (see Appendix A and Figure 1G) for 24 h (Figure 1H). As reported concentrations varied substantially, we stratified studies by concentration range and highlighted the most effective concentration (‘*’) when multiple concentrations were used (Figure 1I and Appendix A). When compared with physiological concentrations (Appendix B), we used much higher concentrations than known human blood levels of these nutraceuticals, except for vitamin E [87]. While these concentrations are far below the reported half-maximal inhibitory concentration (IC50) [88], we must consider the difference in concentrations when interpreting the results.

We found ~75% of studies assessed steatosis qualitatively by immunohistochemical or immunofluorescent staining for neutral lipids or by biochemical quantification of intracellular triglycerides (Figure 1J). However, ~25% of studies reported anti-steatotic effects on transcriptional and/or protein level but did not quantify lipid accumulation (‘none’). Collectively, the wide variability in experimental design, including cell line selection, steatosis induction, and treatment conditions, precludes direct comparison and limits the interpretability of published findings regarding nutraceutical efficacy.

### 3.2. Development of a Standardized in Vitro Assay to Evaluate Anti-Steatotic Effects of Nutraceuticals

To address these inconsistencies across studies, we developed a standardized in vitro assay to evaluate the anti-steatotic effects of nutraceuticals. For this, we selected two cell lines. The HepG2 cancer-derived cell line was chosen because this is the most common cell line used (51 out of 55 studies, Figure 1B). In addition, we selected the Fa2N-4 cell line, which represents an immortalized hepatocyte line derived from a healthy human liver [79,89]. Using these two hepatocyte cell lines, we tested the anti-steatotic effect of eight nutraceuticals (two concentrations for each nutraceutical) under both preventive and therapeutic conditions by assessing intracellular triglyceride accumulation.

**Figure 2:**
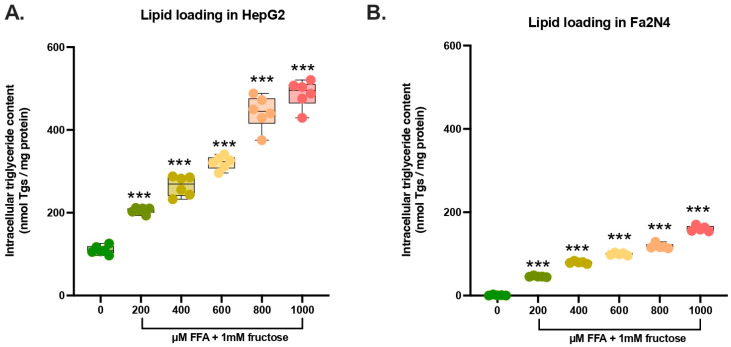
Hepatocyte cell lines HepG2 and Fa2N-4 accumulate lipids. (**A**) HepG2 and (**B**) Fa2N-4 cells stimulated for 24 h with BSA-conjugated free fatty acids (oleic acid and palmitic acid in a 2:1 ratio) with 1 mM fructose. All conditions include n = 5 technical replicates. *** *p* ≤ 0.001.

Steatosis was induced in a dose-dependent manner by exposing HepG2 and Fa2N-4 cells to a 1:2 mixture of FFA (saturated palmitic acid—unsaturated oleic acid) and 1 mM fructose, which closely mimics dietary conditions and aligns with current consensus in the field [90,91]. Basal intracellular triglyceride levels were higher in HepG2 cells than in Fa2N-4 cells (110 nmol triglycerides/mg protein vs. 0.8 nmol triglycerides/mg protein, respectively) (Figure 2). Exposure to FFA + fructose increased triglyceride levels in both cell lines in a dose-dependent manner. For subsequent nutraceutical experiments, we selected a moderate dose of 600 μM FFA + fructose. At this concentration, triglyceride levels increased approximately threefold in HepG2 cells and 179-fold in Fa2N-4 cells. This intermediate fat accumulation provides a suitable range for analyzing both preventive and therapeutic effects, allowing detection of subtle increases or decreases in intracellular triglyceride content.

### 3.3. Anti-Steatotic Effects of Pharmaceuticals

**Figure 3:**
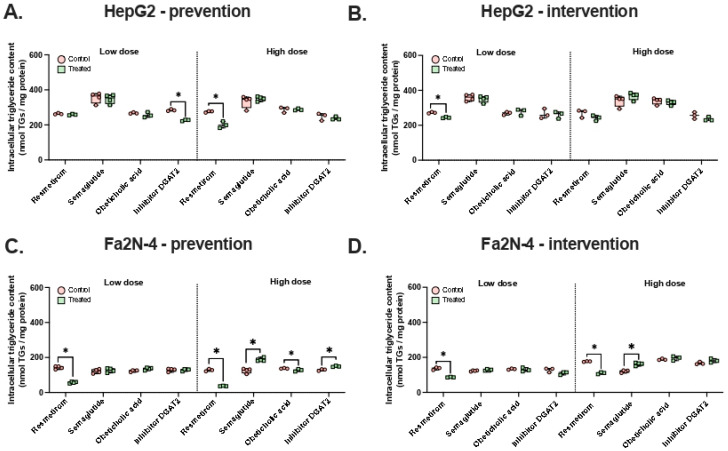
Changes in intracellular triglyceride levels in response to prevention or intervention drug treatment. (**A**) HepG2 cells treated with free fatty acids (FFA) and fructose together with pharmaceuticals at varying concentrations for 48 h as a preventive treatment strategy. (**B**) HepG2 cells treated with FFA and fructose for 24 h, after which pharmaceuticals at varying concentrations are added for another 24 h as an interventive treatment strategy. (**C**) Fa2N-4 cells treated with FFA and fructose, and preventive with pharmaceuticals. (**D**) Fa2N-4 cells treated with FFA and fructose, and intervented with pharmaceuticals. All experiments include n ≥ 3 technical replicates. * *p* ≤ 0.05.

To validate our assay, we first assessed the preventive and therapeutic anti-steatotic effects of four pharmaceutical compounds: the US FDA-approved drugs resmetirom and semaglutide, a DGAT2 inhibitor (PF 06424439), and obeticholic acid [9,90,92,93]. For the preventive experiments, both cell lines were treated with each compound for 48 h simultaneously with FFA and fructose stimuli. For the therapeutic (intervention) experiments, cells were first exposed to FFA and fructose for 24 h, then treated with the pharmaceuticals for an additional 24 h (Figure 3). In each experiment, compounds were tested at two concentrations.

Resmetirom consistently reduced intracellular triglycerides in both preventive and therapeutic experiments across both cell lines. Notably, its therapeutic effect was stronger in Fa2N-4 cells than in HepG2 cells. At a high concentration, resmetirom reduced intracellular triglyceride levels in Fa2N-4 cells by 3.5-fold in the preventive experiment (*p* = 0.05, Figure 3B), compared with only a 1.4-fold reduction in HepG2 cells (*p* = 0.05, Figure 3A). The other pharmaceuticals showed more variable effects depending on the concentration and treatment approach. For example, the DGAT2 inhibitor slightly reduced triglycerides in HepG2 cells at a low concentration (1.3-fold reduction, *p* = 0.05, Figure 3A), while obeticholic acid had a modest protective effect in Fa2N-4 cells at a high concentration (1.1-fold reduction, *p* = 0.046, Figure 3C). However, results were sometimes inconsistent or even contradictory. For instance, a high concentration of the DGAT2 inhibitor increased triglyceride levels in Fa2N-4 cells (1.2-fold, *p* = 0.05, Figure 3C), whereas a low concentration reduced triglyceride levels in HepG2 cells (Figure 3A,B).

These findings indicate that the potential of these compounds for MASLD treatment is highly context-dependent. Nevertheless, the robust effects of resmetirom validate our cellular models and experimental setup, supporting their use for testing the anti-steatotic capacity of nutraceuticals of interest.

### 3.4. Anti-Steatotic Effects of Nutraceuticals

**Figure 4:**
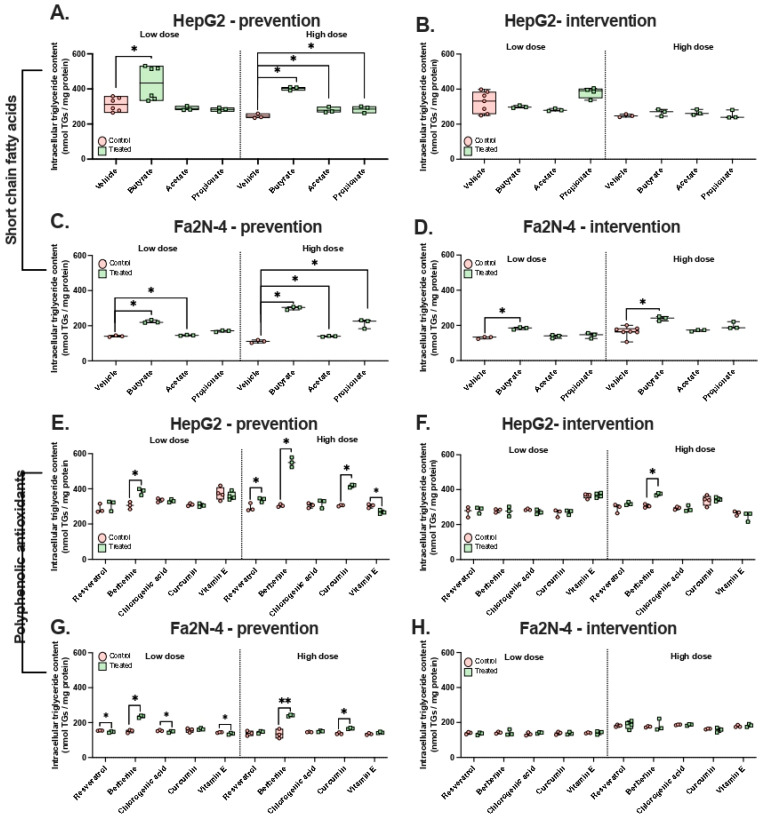
Changes in intracellular triglyceride levels in response to prevention or intervention treatment. (**A**,**E**) HepG2 cells were simultaneously treated with FFA stimuli and nutraceuticals for 48 h (prevention). (**B**,**F**) HepG2 cells were treated with FFA stimuli for 24 h, after which nutraceuticals were added for an additional 24 h (intervention). (**C**,**G**) Fa2N-4 cells were simultaneously treated with FFA stimuli and nutraceuticals for 48 h (prevention). (**D**,**H**) Fa2N-4 cells were treated FFA stimuli for 24 h after which nutraceuticals were added for an additional 24 h (intervention). Short-chain fatty acids were applied at 1 mM (low) and 5 mM (high) concentration. Polyphenolic antioxidants were applied at 10 μM (low) and 50 μM (high). All experiments include n ≥ 3 technical replicates. * *p* ≤ 0.05, ** *p* ≤ 0.01.

We next evaluated the preventive and therapeutic potential of various nutraceuticals using the same experimental setup. Contrary to previous reports suggesting a beneficial role of SCFAs in MASLD, none of the three SCFAs we tested exhibited preventive or therapeutic effects in either cell line, at low or high concentrations (Figure 4A–D). In fact, butyrate, acetate, and propionate all showed pro-steatotic effects under at least one condition. This was particularly pronounced for butyrate, which increased intracellular triglyceride levels in both the preventive (1.7- and 2.7-fold in HepG2 and Fa2N-4 cells, respectively) and therapeutic experiments (1.5-fold increase in Fa2N-4 cells).

The effects of polyphenolic AOXs on triglyceride accumulation were variable, depending on the compound, concentration, and treatment method. No therapeutic effects were observed for any of the polyphenols (Figure 4E–H). Vitamin E demonstrated a modest protective effect in HepG2 cells at a high concentration (1.2-fold decrease, *p* = 0.05, Figure 4E) and in Fa2N-4 cells at a low concentration (1.04-fold decrease, *p* = 0.05, Figure 4G), but had no effect under other conditions. Similarly, low concentrations of chlorogenic acid reduced triglycerides in Fa2N-4 cells by 1.04-fold (*p* = 0.05, Figure 4G).

Resveratrol produced contradictory results. In the preventive experiment, it decreased triglycerides by 1.04-fold in Fa2N-4 cells at a low concentration (*p* = 0.05, Figure 4G), but increased triglycerides by 1.2-fold in HepG2 cells at a high concentration (*p* = 0.05, Figure 4E). In contrast, berberine and curcumin consistently exhibited pro-steatotic effects in multiple conditions, with berberine increasing triglycerides 1.2- to 1.8-fold at high concentrations (except Fa2N-4 intervention, *p* = 0.513) and curcumin causing a 1.2- to 1.4-fold increase in preventive treatments (*p* = 0.05, Figure 4E,G).

Overall, we observed conflicting effects of nutraceuticals on intracellular triglycerides. All the SCFAs, including butyrate, increased triglyceride levels when used as a preventive treatment. Polyphenolic AOX compounds such as berberine and curcumin were surprisingly pro-steatotic under preventive conditions in both cell lines. Vitamin E showed the most consistent protective potential, but at different concentrations in the two cell lines (at a high concentration in HepG2 cells and at a low concentration in Fa2N-4 cells). Together, these findings emphasize that experimental variables such as cell lines and time of administration affect the anti-steatotic potential of nutraceuticals.

## 4. Discussion

To evaluate the anti-steatotic potential of nutraceuticals, standardized validation methods are essential, yet such standardization has been lacking in previous studies. To address this gap, we developed an in vitro assay to quantify intracellular triglyceride accumulation in two hepatocyte cell lines, HepG2 and Fa2N-4. Surprisingly, vitamin E, chlorogenic acid, and resveratrol induced only modest reductions in intracellular triglyceride levels in particular experimental conditions. In addition, several compounds—including SCFAs, berberine, and curcumin—elicited more pronounced pro-steatotic effects.

The pro-steatotic effects of SCFAs, particularly butyrate, contrast with results from the previous literature on in vivo rodents showing decreased liver triglycerides [17,29,94]. On the other hand, in vitro studies reported no change in intracellular triglycerides upon treatment with SCFAs, either in HepG2 cells [29] or in precision-cut liver slices from mice [95]. Previous studies have described SCFAs as anti-steatotic agents [28] or neutral in their effects on steatosis [29,95]. The pro-steatotic activity observed in our study may reflect intrinsic limitations of in vitro 2D cell lines, which lack physiological interactions with organs, particularly with the gut. Supporting this notion, co-culture models combining gut-derived Caco2 cells and HepG2 hepatocytes have shown that butyrate can reduce lipid accumulation [14]. As a mixture of enterocytes and colonocytes, Caco2 cells could metabolize up to 70–90% of butyrate, meaning fatty acid β oxidation metabolites, e.g., ATP and cAMP, could be the main effector molecules in the liver as opposed to whole butyrate, to stimulate the AMPK pathway [17,96].

Furthermore, in vivo, inter-organ crosstalk—such as enhanced adipose tissue lipolysis, an improved gut barrier, and modulation of satiety hormones—may further mitigate hepatic steatosis during butyrate supplementation [17,97,98], which could explain the absence of lipid reduction in our cell-based model. Hepatocytes take up SCFAs mainly by passive diffusion and to a lower extent by MCT1-mediated proton-coupled transport (SLC16A1) [99,100]. Intracellularly, SCFAs are converted to acetyl-CoA, which is a substrate for the citric acid cycle and a precursor for fatty acid synthesis [101]. However, under high-fat feeding, such as our in vitro condition, SCFAs can also be activated to acyl-CoAs, e.g., butyrate to butyryl-CoA, and subsequently be incorporated into triglycerides by esterification of diacylglyceride and butyryl-CoA by the enzyme DGAT1/2 [102]. Our applied concentration, derived from the literature, is much higher than physiological concentrations of SCFAs. Therefore, the administration of excessive SCFA concentrations may well explain our results of exacerbated lipid accumulation as a high-fat feeding state. Future investigation of lipogenesis markers such as DGAT activity and SREP1c protein levels would be needed to confirm this hypothesis.

In contrast to SCFAs, AOXs displayed variable effects on hepatic steatosis in our experiments, ranging from a mild decrease to no effect or even an increase in intracellular triglyceride content. We found a mildly significant decrease (1.04- to 1.2-fold change) in intracellular triglycerides for vitamin E in both cell lines; however, this was not consistent across the dosages, treatment strategies, or cell lines. Notably, both berberine and curcumin induced substantial triglyceride accumulation in HepG2 and Fa2N-4 cells—an observation not previously reported in the context of hepatic steatosis. Both were used at much higher concentrations than in vivo metabolically measured levels in human blood, whereas this was not the case for vitamin E, which was found to be in range [88]. Vitamin E may have exhibited other anti-steatotic or hepatoprotective effects including a reduction in serum aminotransferases, as reported in supplementation human trials [103]. The use of synthetic rather than natural AOX analogs may partly explain the limited anti-steatotic efficacy observed, consistent with a systematic review reporting 100% efficacy for natural AOXs compared to 87.7% for synthetic analogs in animal studies [104]. However, this factor alone does not account for the triglyceride accumulation induced by berberine and curcumin. Since our analysis was limited to intracellular triglyceride quantification, other potentially relevant mechanisms—such as alterations on gene expression levels in lipogenesis, lipolysis, fatty acid oxidation, and antioxidant activity remain unexamined in our study, yet explored in previous cited investigations [32,34,48,54,63,105]. Likewise, possible anti-inflammatory effects, such as secreted cytokines TNFα, IL1β and Il6 [57], gut barrier protection in terms of tight junction proteins [105], and anti-fibrotic effects by a reduction in ECM and collagen [65,95], were not assessed. Therefore, future investigations should aim to explore both the molecular mechanism of fatty acid oxidation and lipogenesis markers, oxidative, ER, and mitochondrial stress markers, while also measuring the consequences on intracellular triglycerides to conclude definitive anti-steatotic effects.

The pro-steatotic effects observed with both SCFAs and OAXs highlight both the relevance and the limitations of this study: anti-steatotic molecular mechanisms do not automatically translate into a reduction in triglyceride levels, but may even increase them. By utilizing a single-endpoint measurement, we can only speculate about the underlying mechanism by which these nutraceuticals increased intracellular triglyceride levels. The utilization of SCFAs as energy substrates is an obvious reason and thus the primary suspect, but for AOXs, berberine, and curcumin, this is not quite as directly deducible. In isolated mitochondria [106], curcumin has a dose-dependent uncoupling effect within the range that we tested the anti-steatotic effect of curcumin (10–50 µM), with mild uncoupling at 25 µM and more severe at 75–100 µM. As a result, there is reduced ATP availability, which may lead to inefficient fatty acid oxidation [106]. Similarly, berberine has also been found to induce oxidative stress by severe mitochondrial uncoupling as well as fragmentation in isolated mitochondria at different concentrations from 33.3, 66.7 and 133.3 ng/mL, similar to the concentrations we used [107]. Thus, AOXs at the concentrations that we used may have disturbed the mitochondria, leading to inadequate fatty acid oxidation.

We also observed that the experimental design, the treatment approach and the concentration of the treatment influenced the outcome. Preventive co-incubation of compounds with FFA and fructose resulted in more pronounced changes in lipid accumulation compared with shorter post-induction interventions in our study. Most nutrient-derived functional compounds are known for their preventive effects, while their intervention potential is currently still being investigated [108,109,110]. We also noticed that there was no specific trend depending on the concentration, except for a dose-dependent increase upon pro-steatotic effects. A different treatment concentration of the same compound may have differential effects, as a recent review on resveratrol noted a dual dose-dependent effect: a preventive, antioxidant effect at a lower dose, whereas intervention at a high dose had notable anti-cancerous properties [111]. In our case, we used resveratrol at a high concentration. It may therefore not have functioned to elevate oxidative stress resulting in lipid breakdown. To our knowledge, this is the first exploration that clearly distinguishes side-by-side the prevention vs. therapeutic effects of nutraceuticals solely based on triglyceride levels. We wish to highlight the importance of clearly defining treatment strategies in screening studies, as the literature rarely distinguishes between preventive and therapeutic setups, despite their potential impact on results.

To validate our assay, we included several pharmaceutical compounds—resmetirom, semaglutide, obeticholic acid, and a DGAT2 inhibitor. Resmetirom markedly reduced triglyceride levels in both HepG2 and Fa2N-4 cells. The other pharmaceutical agents induced only minor changes in intracellular triglycerides, suggesting that in vitro anti-steatotic effects are not easily captured in our simplified system, despite strong evidence from animal and clinical studies [10,73,93,112]. Semaglutide, a GLP-1 receptor agonist, primarily acts on the intestine, pancreas and brain, indirectly reducing hepatic steatosis through weight loss rather than direct hepatic mechanisms [11,76]. Nevertheless, we evaluated semaglutide in HepG2 and Fa2N-4 cells to determine whether it also elicits cell-autonomous effects on triglyceride handling, independent of its systemic actions. However, we did not observe anti-steatotic effects of resmetirom on isolated hepatocytes.

Obeticholic acid, a potent FXR agonist, acts directly on hepatocytes to modulate bile acid signaling and lipid metabolism. Obeticholic acid showed promise for treating nonalcoholic steatohepatitis, but it was recently rejected by both the US FDA and EMA due to adverse hepatic outcomes [66,113]. Despite its direct hepatic mechanism of action, we could not find a clear anti-steatotic effect in HepG2 and Fa2N-4 cells, which might be due to a lack of FXR signaling. Lastly, we tested a DGAT2 inhibitor (PF-06424439) and found that it did not change intracellular triglyceride levels in lipid-loaded hepatocytes. DGAT2 inhibitors target diacylglycerol O-acyltransferase 2, a key enzyme catalyzing the final step in triglyceride synthesis. Because DGAT2 acts directly within hepatocytes to regulate intracellular triglyceride production, these compounds are currently under clinical investigation as potential therapeutics for steatotic liver disease [78,93]. Specifically, PF-06424439 is still under preclinical investigation and shows slow, reversible, time-dependent inhibition of DGAT2 [114]. Previous reports on HepG2 [93] or primary hepatocytes from mice and primates [70] under steatotic stimuli only show an effective reduction in triglycerides upon dual inhibition of DGAT1 and DGAT2. DGAT1 may compensate for the absence or reduced activation of DGAT2 to synthesize triacylglycerol in human primary hepatocytes [115].

Differences in culture conditions can also cause variability across studies. Many reports do not specify basal media composition or glucose concentration, parameters that are critical for metabolic studies. For example, commonly used media contain supraphysiological glucose levels of 11 mM (e.g., Williams’ E, RPMI) or 25 mM (e.g., DMEM), compared to physiological plasma levels of 3.9–7.8 mM [116]. In our study, we maintained standard culture conditions to reproduce prior findings without altering glucose levels, serum content, or insulin supplementation. However, these factors likely influence the degree of steatosis induction and the apparent treatment response. Notably, the only publicly available formulation for Fa2N-4 cells, described by Padberg et al. (2021), includes insulin [117], which, combined with the different glucose concentrations between DMEM and Williams’ E medium, may have contributed to the unexpected pro-steatotic effects observed and the limited reproducibility across studies. Next, approximately 25% of reviewed studies reported no change in intracellular lipid accumulation, neither biochemically nor histologically. Given the potential for bias in qualitative lipid staining, we employed a quantitative whole-cell lipid extraction and intracellular triglyceride assay [118,119], which we consider a more objective and reproducible readout for assessing steatosis. Multiple previous nutraceutical studies reviewed do not show a change in neutral lipid levels despite anti-MASLD molecular adaptations [29,43,95]. However, we limited our investigation to a single-endpoint measurement and thus do not provide mechanistic insight into whether the nutraceuticals (or pharmaceuticals) affected lipid metabolism or other MASLD features such as oxidative stress and inflammation.

A key limitation of our study is the focus on a single endpoint—intracellular lipid content—in only two hepatocyte cell lines. It is possible that early anti-steatotic mechanisms, such as activation of fatty acid oxidation pathways (e.g., CPT1A–AMPKα1–ACC signaling), occurred without translating into measurable reductions in triglyceride levels. Additionally, we did not assess other MASLD-related processes, including oxidative stress, inflammation, or fibrosis. Including the investigation of molecular mechanisms and MASLD-related processes using a standardized assay in future studies will enhance our knowledge of the anti-steatotic effects of nutraceuticals.

## 5. Conclusions

Our findings underscore the importance of standardized assays in drug screening within disease-relevant models. The comparison of preventive and therapeutic in vitro treatment strategies revealed variable outcomes, emphasizing the need for further investigation in a standardized setting. Unexpectedly, several nutraceuticals previously reported as being anti-steatotic—butyrate, berberine, and curcumin—exhibited pro-steatotic effects in our hepatocyte models. These contradictory results highlight the critical need for transparency and reproducibility in in vitro research and may help explain the limited clinical success of certain nutraceutical interventions [108].

Finally, our study demonstrates the use of intracellular triglyceride quantification as a practical and informative endpoint for anti-steatotic compound screening. Establishing a clear in vitro anti-steatotic effect in hepatocytes should be considered a fundamental preclinical requirement before advancing candidates into clinical development. Incorporating this approach could strengthen the translational pipeline for nutrient-derived therapeutics targeting MASLD.

## Figures and Tables

**Table 1:** Overview of the literature on selected nutraceuticals that improve metabolic diseases and show mechanisms of protection in MASLD.

Nutraceutical Class	Compound	Main Functions in Metabolic Diseases	Mechanisms of MASLD Protection
Nutrients: short-chain fatty acids (SCFAs)	Butyrate	Plays a vital role in intestinal homeostasis, serving as an energy source and exhibiting anti-inflammatory properties that contribute to intestinal barrier function and immunity [26].	Exhibits anti-steatotic properties by suppressing lipogenesis via inhibition of SREBP-1c and its target genes SCD1 and FAS and inducing fatty acid oxidation via suppression of PPARα and subsequent upregulation of the UCP2-pAMPK-pACC pathway. Butyrate also modulates hepatic GLP-1 receptor expression and contributes to regulation of glucose and insulin homeostasis and appetite suppression [14,17,28,29,30,31].
	Acetate	Plays a role in appetite control in the brain and serves as a major energy supplier [26].	Upregulates fatty acid oxidation via PPARα and the UCP2-pAMPK-pACC pathway and downregulates lipolysis via GPR43. In vitro studies demonstrate anti-tumorigenic capacity by inhibiting cell growth and suppressing the IL-6-JAK1-STAT3 pathway, as well as inhibiting expression of the cancer gene MYC [17,32].
	Propionate	Inhibits hepatic fatty acid production and serves as a precursor for gluconeogenesis [26].	Regulates fatty acid oxidation via PPARα, CPT1A, and UCP2 under conditions of oxidative stress [17,30].
Phytochemicals: polyphenolic antioxidants (AOXs)	Resveratrol	Has antioxidant and anti-inflammatory capacities, with subsequent implications in metabolic diseases [33].	Anti-steatotic by increasing mitochondrial activity (via SIRT1 and ATP storage) and decreasing damaging ROS, increasing fatty acid oxidation (via CPT1A, AMPKα1-ACC, and/or SREBP-1C expression) and autophagy (ATG5) [22,23,34,35,36,37,38,39,40,41,42,43,44].
	Curcumin	Has antioxidant and anti-inflammatory capacities, with subsequent implications in metabolic diseases [33,45].	Anti-steatotic by increasing fatty acid oxidation (CPT1A), downregulating lipid and cholesterol metabolism and transport (FABP1, APOC3, and GK), and reducing intracellular-damaging ROS [22,43,46,47,48,49,50].
	Berberine	Most known for its anti-bacterial and anti-inflammatory properties [51].	Anti-steatotic by upregulation of fatty acid oxidation via CPT1A expression and by suppression of lipogenesis via inhibition of ACC1 and FAS and reduction in intracellular damage via ROS. It also improves glucose homeostasis via increased glucose uptake along with glycogen synthesis and suppression of gluconeogenesis [14,22,43,52,53,54,55].
	Chlorogenic acid	Has antioxidant properties and is preventive or/and protective in metabolic syndrome such as obesity and dyslipidemia [56].	Anti-steatotic by decreasing lipogenesis (ACC, FAS) and increasing fatty acid oxidation (CPT1) via AMPK phosphorylation [57,58,59,60,61].
	Vitamin E (α-tocopherol)	Has antioxidant properties and is used as off-label therapy in non-diabetic MASLD patients [24,62].	Anti-steatotic by decreasing lipogenesis (ACC, FAS) and increasing fatty acid oxidation (CPT1) via AMPK phosphorylation [49,63,64,65].

SCD1, stearoyl-CoA desaturase 1; FAS, fatty acid synthase; SREBP-1c, sterol regulatory element binding protein 1c; PPARα, peroxisome proliferator-activated receptor (PPAR)-alpha; UCP2, mitochondrial uncoupling protein 2; pAMPK, phosphorylated AMP-activated protein kinase; pACC, phosphorylated acetyl-CoA carboxylase; GLP-1, glucagon-like peptide 1; IL-6, interleukin 6; JAK1, Janus kinase 1; STAT3, signal transducer and activator of transcription 3; CPT1A, carnitine palmitoyltransferase 1A; ATG5, autophagy protein 5; SIRT1, sirtuin 1; FABP1, fatty acid binding protein 1; APOC3, apolipoprotein C3; GK, glycerol kinase; ROS, reactive oxygen species.

**Table 2:** Pharmaceutical drugs investigated in the context of MASLD. FXR, Farnesoid X receptor; GLP-1, glucagon-like peptide 1; THRB, thyroid hormone beta receptor; DGAT2, diacylglycerol O-acyltransferase 2.

Compound	Status in Drug Investigations	Mechanisms of MASLD Protection
Obeticholic acid; INT-747	Rejected [72]	FXR agonist that regulates bile, cholesterol, and lipid metabolism. Has been found to improve blood glucose levels and hepatic fibrosis in clinical trials. Reported to reduce hepatic steatosis, ballooning, and lobular inflammation [67,73].
Resmetirom; MGL-3196	Conditionally approved [8]	THRB agonist that stimulates mitochondrial beta-oxidation, both directly and indirectly via transcription factors. Moreover, it promotes hydrolyzation of lipid droplets, instigates lipophagy, inhibits inflammatory signals [74], and results in significant resolution of steatohepatitis and fibrosis [10].
Semaglutide; NN-9535	Approved [9]	GLP-1 analog that stimulates pancreatic beta cells for glucose-dependent insulin secretion, suppresses glucagon secretion, delays gastric emptying, and reduces food intake via leptin signaling. This leads to weight loss and metabolic improvements, including decreased dietary fat intake and export to the liver, increased insulin sensitivity, reduced de novo lipogenesis, and reduced inflammation [75,76].
Inhibitor for DGAT2; PF-06424439	Preclinical phase [77]	Selective, potent DGAT2 inhibitor that suppresses synthesis of lipids, leading to reduced triglyceride accumulation and secretion from the liver in rodents [71]. Shows effective reduction in steatosis and improved liver function in healthy human individuals [78].

**Table 3:** Therapeutics, solvents, and concentrations.

Therapeutics	Company, Catalog #	Solvent	Concentration on Cells ^1^
Sodium butyrate	Sigma Aldrich, 303410	PBS (1×)	1 mM or 5 mM
Sodium acetate	Sigma Aldrich, S8750	PBS (1×)	1 mM or 5 mM
Sodium propionate	Sigma Aldrich, P1880	PBS (1×)	1 mM or 5 mM
Sodium chloride	Sigma Aldrich, 106.404	PBS (1×)	1 mM or 5 mM
Resveratrol	Sigma Aldrich, 554325	DMSO	10 µM or 50 µM
Curcumin	Sigma Aldrich, C7727	DMSO	10 µM or 50 µM
Berberine	Sigma Aldrich, B3412	Methanol:H_2_O (2:1)	10 µM or 50 µM
Chlorogenic acid	Sigma Aldrich, C3878	Ethanol	10 µM or 50 µM
Vitamin E (α-tocopherol)	Sigma Aldrich, V-020	Methanol	10 µM or 50 µM
Resmetirom (MGL-3196)	Axon Medchem ^2^, 2657	DMSO	100 µM or 200 µM [80]
Obeticholic acid (INT-747)	Selleckchem ^3^, 501365091	DMSO	1 µM or 10 µM [68]
Selective DGAT2 inhibitor (PF 06424439)	Tocris ^4^, 6348	DNase/RNase-free H_2_O	2.5 µM or 10 µM [69,77]
Semaglutide (injection pen, 1.34 mg/mL)	Novo Nordisk ^5^, EAN 8717371986162	Sterile H_2_O	1 µM or 10 µM [81]

# indicates catalog number. ^1^ The solvents were used as controls for the corresponding treatments and did not exceed 0.8% (*v*/*v*) to limit toxicity. ^2^ Groningen, The Netherlands ^3^ Houston, TX, USA ^4^ Bristol, UK ^5^ Bagsværd, Denmark.

## Data Availability

The original contributions presented in this study are included in the article. Further inquiries can be directed to the corresponding author.

## Abbreviations

The following abbreviations are used in this manuscript:

ACC	Acetyl-CoA carboxylase
AMPKα1	AMP-activated catalytic subunit alpha 1
AOXs	Antioxidants
CPT1A	Carnitine palmitoyltransferase I
DGAT2	Diacylglycerol O-acyltransferase 2
DMEM	Dulbecco’s modified eagle media
EMA	European Medicines Agency
FDA	Food and Drug Administration USA
FFA	Free fatty acids
HDAC	Histone deacetylase
MASH	Metabolically associated steatohepatitis
MASLD	Metabolically associated steatotic liver disease
PPARα	Peroxisome proliferator-activated receptor alpha
RPMI	Roswell Park Memorial Institute media
SCFAs	Short-chain fatty acids
SREBP-1c	Sterol regulatory element binding protein-1c

## Appendix A

**Table A1:** Overview of in vitro data on the effects of various nutraceuticals in human hepatocytes. If multiple concentrations were used, * indicates the optimal condition per the authors. Abbreviations: 4-HNE, 4-hydroxynonenal; ACC, Acetyl-CoA Carboxylase; ALKBH5, AlkB Homolog 5 RNA Demethylase; ALT, Alanine Transaminase; AMPK, AMP-activated protein kinase; AST, Aspartate transaminase; BODIPY, 4,4-difluoro-4-bora-3a,4a-diaza-s-indacene; CCK-8, Cell Counting Kit-8; CPT, Carnitine Palmitoyltransferase; COL1A1, Collagen type I, alpha 1; DCF-DA, 2′,7′-dichlorodihydrofluorescein diacetate; FACS, Fluorescence-Activated Cell Sorting; FTO, FTO Alpha-Ketoglutarate Dependent Dioxygenase; JC-1, 5,5′,6,6′-Tetrachloro-1,1′,3,3′-tetraethylbenzimidazolylcarbocyanine iodide; MTT, 3-(4,5-dimethylthiazol-2-yl)-2,5-diphenyltetrazolium bromide; NAPDH, Nicotinamide adenine dinucleotide phosphate reduced; NRF2, Nuclear factor erythroid 2-related factor 2; PCR, Polymerase Chain Reaction; ROS, reactive oxygen species; SIRT1, Sirtuin-1; SOD, Superoxide dismutase; TMRE, Tetramethylrhodamine ethyl ester).

Nutraceutical Class	Compound	MASLD Model Cell Line, Basal Media, Stimulations	Nutraceutical Treatment Concentration, Time Duration	Assays Technique, Specific Assays	Reference
Probiotic micro-organism (microbiome-derived metabolites)	Butyrate	HepG2 cultured in DMEM/F12 with regular glucose (17.5 mM). Prior to stimulation, serum starved for 24 h. Thereafter, stimulated for another 24 h with higher glucose (33 mM) and insulin (100 nM)	Co-treatment for 24 h with 1, 2, 5 * or 10 * mM butyrate	Western blot, neutral lipid staining (Oil Red O, BODIPY), intracellular triglyceride quantification	[28]
		HepG2 cultured in DMEM (25 mM glucose)	Treatment for 24 h with 3 mM butyrate	Quantitative PCR (QPCR), Western blot	[17]
		HepG2 cultured in DMEM (5.5 mM glucose) and two steatosis models: 1 mM oleic acid/palmitic acid * for 24 h and 1 µg/mL LPS for 24 h	Post-treatment for 24 h with 2 *, 4 * or 8 mM butyrate	QPCR, enzyme-linked immunosorbent assay	[30]
		Co-culture HepG2 and Caco2 cells in Transwells or microfluidic chip in DMEM (undefined high glucose) and stimulated with 1.2 mM palmitic acid for 24 h	Co-treatment for 24 h with 2 mM butyrate	Immunofluorescent (IF) antioxidant staining (DCF-DA)	[105]
		HepG2 cultured in RPMI 1640 (5.5 mM, 1 g/L glucose), partial serum-starvation (2%) for 6 h and treated with 2 mM valproate (anti-epileptic drug) for 48 h	Pre-treatment for 1 h with 0.5 or 1 mM butyrate	Antioxidant assay (DCFH), lipid peroxidation (malondialdehyde concentration), Western blot, neutral lipid staining (Oil Red O), mitochondrial function (SOD, CPT, aconitase activity), cellular oxygen consumption (Seahorse)	[31]
		HepG2 cultured in DMEM (undefined glucose concentration) and stimulated with 0.5 mM FFA mix of oleic and palmitic acid (2:1) for 24 h	Co-treatment for 24 h with 1, 2, 5 * and 10 mM butyrate	Intracellular triglyceride concentration, Western blot	[29]
	Acetate	HepG2 cultured in DMEM (25 mM glucose)	Treatment for 24 h with 3 mM acetate	QPCR, Western blot	[17]
		HKCI2 and HKCI10 (human NAFLD-HCC cell lines) cultured in RPMI 1640 (11 mM glucose)	Treatment for 24 *−72 h with 10% of sodium acetate	Cell viability (MTT) and apoptosis (FACS), cell colony formation (crystal violet staining), Western blot	[32]
	Propionate	HepG2 cultured in DMEM (5.5 mM glucose) and two steatosis models: (1) 1 mM oleic acid/palmitic acid * for 24 h or (2) 1 µg/mL LPS for 24 h	Post-treatment for 24 h with 2, 4 * or 8 * mM propionate	QPCR, enzyme-linked immunosorbent assay (ELISA)	[30]
		HepG2 cultured in DMEM (25 mM glucose)	Treatment for 24 h with 3 mM propionate	QPCR, Western blot	[17]
Chemical compounds (Plant nutrients)	Resveratrol	HepG2 cultured in DMEM (5.5 mM glucose) and stimulated with oleic acid (1.5 mM) for 24 h	Pre-treatment for 2 h with 10 µM resveratrol	Neutral lipid staining (Nile Red), mitochondrial membrane potential staining (TMRE), QPCR, Western blot, SIRT1 deacetylase activity, intracellular ATP quantification (CellTiter-Glo Luminescent, Cell Viability Assay kit)	[22]
		HepG2s cultured in DMEM (5.5 mM glucose) and stimulated with higher glucose (33 mM) for 48 h	Co-treatment for 48 h with 20 µM resveratrol	Cell viability (MTT), quantitative methylation-specific PCR, Western blot, QPCR	[23]
		HepG2 cultured in DMEM (25 mM glucose) and stimulated for 24 h with 0.2 mM palmitic acid	Post-treatment for 24 h with 40 µM resveratrol	Neutral lipid staining (Oil Red O)	[35]
		HepG2 cultured in EMEM (minimal essential medium, 5.5 mM glucose) and stimulated for 24 h with oleic acid (0.1–0.2 mM), palmitic acid (0.1–0.2 mM) or combined at 2:1 ratio (0.1–0.2 mM)	Co-treatment for 24 h with 10 or 20 µM resveratrol	Neutral lipid staining (Nile Red), cell viability (MTT), mitochondrial membrane potential (fluorescent dye JC-1), IF staining for oxidative stress (MitoTracker, RedoxSensor)	[38]
		HepG2 cultured in DMEM (undefined glucose concentration) and treated with 0.2 mM palmitic acid for 24 h	Post-treatment for 24 h with 10, 20 or 40 µM resveratrol	Cell viability (CCK-8), neutral lipid staining (Bodipy), intracellular triglyceride accumulation	[37]
		HepG2 cultured in DMEM (5.5 mM glucose), incubated overnight serum-free media incubation and stimulated with higher glucose (30 mM) for 24 h	Post-treatment for 1 h with 10 µM resveratrol	Cellular ATP levels (ATPLite), immunoprecipitated and immunoblotting, intracellular triglyceride and cholesterol concentration	[120]
		HepG2 cultured in DMEM (5.5 mM glucose), incubated overnight serum-free media incubation and stimulated with higher glucose (30 mM) for 24 h	Co-treatment for 24 h with 1, 10, 50 * µM resveratrol	SIRT1 fluorescence assay, immunoblot analysis, intracellular triglyceride concentration, SIRT1 lentivirus-mediated knockdown	[42]
		HepG2 cultured in DMEM (5.5 mM glucose), incubated overnight serum-free media incubation and stimulated with higher glucose (25 mM) and insulin (100 nM) for 24 h	Co-treatment for 24 h with 50 µM resveratrol	Neutral lipid staining (Oil Red O), intracellular triglyceride concentration, Western blot, real-time PCR (RT-PCR)	[41]
		HepG2 cultured in DMEM (undefined glucose concentration) and treated 0.2 mM palmitic acid for 24 h	Post-treatment for 24 h with 20, 40 * or 80 µM resveratrol	Neutral lipid staining (Oil Red O), quantification transmission electron microscopy, IF staining for autophagy and lysosomes, Western blot, SIRT1 activity assay	[35]
		HepG2 cultured in DMEM (undefined glucose concentration) and treated 0.1 mM oleic acid and 87 mM alcohol for 48 h	Co-treatment for 48 h with 5, 15, 45 * or 135 µM resveratrol	Neutral lipid staining (Oil Red O), intracellular triglyceride concentration, Western blot	[40]
	Resveratrol				
		HepG2 cultured in DMEM (undefined glucose concentration) and treated with 2 mM palmitic acid for 24 h	Co-treatment with 40 µM resveratrol for 24 h	Cell viability (MTT), neutral lipid staining (Oil Red O), RT-PCR, IF staining, Western blot	[39]
		HepG2 cultured in DMEM (undefined glucose concentration) and treated with 1 mM oleic acid for 24 h	Co-treatment with 100 µM resveratrol for 24 h	Neutral lipid staining (Oil Red O), intracellular triglyceride concentration	[36]
		HepG2 cultured in DMEM (undefined high glucose) and treated with 0.24 mM oleic acid for 24 h	Co-treatment with 12.5, 25, 50 or 100 µM resveratrol for 24 h	Neutral lipid staining (Oil Red O), intracellular triglyceride and glycerol concentration, cell viability (MTT)	[34]
		HepG2 cultured in EMEM (5.5 mM glucose) and post-treated for 24 h with 1.5 mM oleic acid	Pre-treatment for 2 h with 1, 5 or 10 * μM resveratrol	Intracellular ROS, mitochondrial content (MitoTracker), QPCR	[43]
		HepG2 cultured in DMEM (undefined glucose concentration) and stimulated with 0.5 mM palmitic acid and 30 mM glucose for 48 h	Post-treatment for 20 min, 6 h or 24 h * with 1, 5, 10 * or 50 µM resveratrol. Afterwards 16 h FBS starvation and exposed to 10 µM leptin for 20 min	Cell viability (neutral red assay), intracellular triglyceride concentration, Western blot, ultra-high-performance LS-MS for resveratrol metabolite (RSV-3-sulfate), QPCR, SIRT1 activity, IF staining (leptin receptor)	[44]
	Curcumin	HepG2 cultured in DMEM (5.5 mM glucose) and stimulated with oleic acid (1.5 mM) for 24 h	Pre-treatment for 2 h with 10 µM curcumin	Neutral lipid staining (Nile Red), mitochondrial membrane potential staining (TMRE), QPCR, Western blot, SIRT1 deacetylase activity, intracellular ATP quantification (CellTiter-Glo Luminescent Cell Viability Assay kit)	[22]
		HepG2 cultured in DMEM (25 mM glucose) and stimulated with nonylphenol (degradation product from nonylphenol ethoxylate, classified as industrial endocrine disrupting chemical) for 24 h	Co-treatment for 24 h with 5–20 µM curcumin	Cell viability (CCK-8), neutral lipid staining (Nile red), quantitative total triglycerides and cholesterol, QPCR, apoptosis (flow cytometry), Western blot	[46]
		HepG2 cultured in EMEM (minimal essential medium, 5.5. mM glucose) and treated with 0.5 mM oleic acid and 0.25 mM palmitic acid (2:1) for 24 h	Post-treatment for 24 h with 5 µM curcumin alongside steatotic induction (oleic and palmitic mix)	Cell viability (MTT), neutral lipid staining (Oil Red O), IF ROS staining, RT-PCR microarray	[47]
		HepG2 cultured in RPMI 1640 (11 mM glucose) and treated with 0.15 mM oleic acid and 2.5 µg/mL LPS for 24 h	Co-treatment for 24 h with 10 µM curcumin	Neutral lipid staining (Oil Red O), intracellular triglyceride content, flow cytometry and IF staining for ROS, Western blot	[50]
		HepG2 cultured in DMEM (undefined glucose concentration) and treated with 0.5 mM oleic acid for 24 h	Post-treatment for 24 h with 5–20 µM di-hydrocurcumin (major metabolite of curcumin)	Cell viability (MTT), intracellular triglyceride content, QPCR, Western blot	[48]
		HepG2 cultured in EMEM (5.5 mM glucose) and post-treated for 24 h with 1.5 mM oleic acid	Pre-treatment for 2 h with 1, 5 or 10 μM * curcumin	Intracellular ROS, mitochondrial content (MitoTracker), QPCR	[43]
		HepG2 cultured in DMEM (undefined glucose concentration) and treated with 6 mM of a mixture of linoleic acid and oleic (1:1) for 24 h	Post-treatment for 24 h with 0.001, 0.005 * (±13.6 μM) or 0.05 mg/mL curcumin	Neutral lipid staining (Oil Red O), cell viability (MTT), QPCR, Western blot	[49]
	Berberine	HepG2 cultured in DMEM (25 mM glucose) and stimulated with 0.5 mM FFA mix for 24 h	Co-treatment for 24 h with 15.87 μg/mL (47.2 µM) berberine	Neutral lipid staining (Oil Red O), intracellular triglyceride content, cell viability (MTT), fluorescent ROS content	[54]
Chemical compounds (Plant nutrients)		HepG2 cultured in DMEM (5.5 mM glucose) and stimulated with oleic acid (1.5 mM) for 24 h	Pre-treatment for 2 h with 10 µM berberine	Neutral lipid staining (Nile Red), mitochondrial membrane potential staining (TMRE), QPCR, Western blot, SIRT1 deacetylase activity, intracellular ATP quantification (CellTiter-Glo Luminescent Cell Viability Assay kit)	[22]
		HepG2 cultured in DMEM (undefined high glucose) and stimulated with 0.2 mM oleic acid for 24 h	Co-treatment for 24 h with 1.25–5 µM berberrubine (a main active metabolite of berberine)	Cell viability (MTT), neutral lipid staining (Oil Red O), intracellular triglyceride content, glucose uptake assay, Western blot	[52]
		HepG2 cultured in DMEM (undefined glucose concentration) and stimulated with 500 μM FFA mix (2:1 ratio oleic to palmitic acid) for 24 h	Co-treatment for 24 h with 1, 5 or 25 * (±65 μM) μg/mL berberine	Cell viability (CCK-8), neutral lipid staining (Oil Red O), intracellular triglyceride content, QPCR, Western blot, IG staining	[121]
		HepG2 cultured in EMEM (5.5 mM glucose) and post-treated for 24 h with 1.5 mM oleic acid	Pre-treatment for 2 h with 1, 5 or 10 * μM berberine	Intracellular ROS, mitochondrial content (MitoTracker), QPCR	[43]
		Huh7 cultured in DMEM (undefined glucose concentration) and stimulated with FFA (0.3 mM, oleic and palmitic acid in 2:1 ratio) for 8 h	Pre-treated for 24 h with 10 μM berberine	IF staining (Nrf2, Mitosox, DCF-DA), Western blot, QPCR, enzyme activity (SOD), neutral lipid staining (Nile Red), respiration measurement (XF24 analyzer)	[122]
		Huh7 cultured in DMEM (undefined glucose concentration) and stimulated with oleic acid (0.1 mM) for 24 h	Co-treated for 24 h with 10 μM berberine	Neutral lipid staining (Nile Red), QPCR	[123]
		HepG2 cultured in DMEM (undefined glucose concentration) and stimulated 33 mM glucose for 24 h	Pre-treatment for 2 h with 5, 10, 20 or 40 * μM berberine	Neutral lipid staining (Oil Red O), intracellular triglyceride concentration, QPCR	[53]
		HepG2 cultured in RPMI 1640 (11 mM glucose), with lipoprotein-deficient serum and treated for 12 h with 1 nmol/L insulin	Co-treatment for 12 h with 7.5 μg/mL (±20 μM) berberine	Lipid synthesis, phosphorylation of ACC and AMPK measurement, AMPK activity, fatty acid oxidation assay	[55]
Chemical compounds (Plant nutrients)		Co-culture HepG2 and Caco2 cells in Transwells or microfluidic chip in DMEM (high glucose concentration) and stimulated with 1.2 mM palmitic acid for 24 h	Co-treatment for 24 h with 10 μM berberine	IF antioxidant staining (DCF-DA)	[105]
	Chlorogenic acid	HepG2 cultured in DMEM (undefined glucose concentration) and stimulated with 25 mM glucose and 250 μM oleic acid for 24 h	Post-treatment for 24 h with 50 μg/mL (±141 μM) chlorogenic acid	Neutral lipid staining (Oil Red O), intracellular triglycerides and total cholesterol content, QPCR, Western blot, calcium flux and apoptosis (flow cytometry), liver damage markers ALT and AST, fatty acid β-oxidation (colorimetric assay)	[59]
		HepG2 cultured in DMEM (5.5 mM glucose) and stimulated with higher glucose (33 mM) for 24 h	Pre-treatment for 2 h of 5–50 μM chlorogenic acid	Cell viability (MTT), neutral lipid staining (Oil Red O), intracellular triglyceride content, QPCR, Western blot	[58]
		HepG2 cultured in RPMI 1640 (11 mM glucose), before stimulated serum starved for 12 h and stimulated with 0.1 mM oleate	Co-treated for 24 h or 48 h * with 30 μM chlorogenic acid	Cell viability (MTT), neutral lipid staining (Oil Red O), secreted cholesterol and triglyceride concentrations, QPCR	[60]
		HepG2 cultured in DMEM (undefined glucose concentration) with 1 mM FFA (palmitic/oleic acid) for 12, 24 h *, 48 h or 72 h	Post-treatment for 12, 24 h *, 48 h or 72 h with 50 μM chlorogenic acid	QPCR, Western blot, ChIP analysis, neutral lipid staining (Oil Red O), intracellular triglycerides content	[124]
		THLE-2 cultured in DMEM (25 mM glucose) with 2.5% lipid mixture (L0288, Sigma, USA) for 24 h	Post-treatment for 24 h with 25–100 * μM chlorogenic acid	QPCR, demethylase activity (FTO, ALKBH5), Drug Affinity Responsive Target Stability and Cellular Thermal Shift Assay, Western blot, gene of interest knockdown by transduction	[125]
		HepG2 cultured in 0.1% gelatin-coating on DMEM (undefined glucose concentration) and stimulated with 0.4 mM FFAs for 24 h–48 h	Co-treatment of 24 h with 125, 250 or 500 μM chlorogenic acid	Lipid peroxidation (Liperfluo) and neutral lipid IF staining (SRfluor680), LC-MS analysis	[57]
		HepG2 cultured in DMEM (undefined glucose concentration) and stimulated 0.5–1.5 mM of oleic acid for 24 h	Co-treated for 24 h with 20–100 * μg/mL chlorogenic acid	Cell viability (MTT), neutral lipid staining (Oil Red O), ALT and AST levels, IF antioxidant staining (DCF-DA)	[126]
		HepG2 cultured in DMEM (5.5 mM glucose) and stimulated with 0.5 mM palmitic acid for 24 h	Co-treatment of 24 h with 50 μM chlorogenic acid	ELISAs ((p)ERK1/2, FGF21, TNFα, IL-6, IL-1β), Western blot, protein phosphorylation array, cell viability, NOS activity, LDH release, ROS production (DCF-DA), mitochondrial O_2_^•−^ (Mitosox), NAPDH, SOD, catalase oxidase activity, mitochondrial function (Mitotracker), neutral lipid staining (Oil Red O), intracellular triglycerides and glycerol concentration, glucose uptake, glucokinase activity and glucose production	[61]
Chemical compounds (Plant nutrients)	Vitamin E (α-tocopherol)	HepG2 cultured in DMEM (5.5 mM glucose) and stimulated for 48 h with higher glucose media (25 mM)	Co-treatment for 48 h with 100 μM vitamin E	Intracellular triglycerides content, QPCR, isotopic-glucose incorporation in lipids, Western blot, IF staining of SCREBP-1, lipid peroxidation (4-HNE and Click-IT)	[63]
		Three-dimensional spheroids consisting of HepG2 and LX-2 cells in 24:1 ratio. Spheroids are cultured in MEM (5.5 mM glucose) and stimulated with 0.5 mM mix of oleic and palmitic acid (2:1 ratio) for 24–48 h	Co-treatment for 24–48 h with 10–50 μM vitamin E	Neutral lipid staining (AdipoRed), cell viability (CellTiter-Glo Luminescent Cell Viability Assay kit), IF staining collagen (COL1A1)	[65]
		HepG2 cultured in DMEM (5.5 mM glucose), after 24 h serum-starvation, stimulated with higher glucose (25 mM) for 48 h	Co-treatment for 48 h with 25, 50 or 100 * μM vitamin E	Intracellular triglyceride concentration, radioactively labeled glucose incorporation in lipids, QPCR, Western blot, IF staining for lipid peroxidation (4-HNE, Click-IT) and SREBP-1	[63]
		Hepg2 cultured in MEM (5.5 mM glucose) and stimulated with 30 mM fructose, 0.05 mM palmitic acid * and/or 0.05 mM oleic acid * for 24 h	Co-treated for 24 h with 100 μM vitamin E	Neutral lipid staining (Oil Red O), uptake α-tocopherol, γ-tocopherola and α-13′OH metabolite formation, Western blot	[64]
		HepG2 cultured in DMEM (undefined glucose concentration) and treated with 6 mM of a mixture of linoleic acid and oleic (1:1) for 24 h *	Post-treatment for 24 h with 0.001, 0.005 * (±11.6 µM) or 0.05 mg/mL vitamin E	Neutral lipid staining (Oil Red O), cell viability (MTT), QPCR, Western blot	[49]

## Appendix B

**Table A2:** Relevant concentrations of short-chain fatty acids and antioxidant compounds. Summarized are the reported blood levels per compound based on the Human Metabolome Database (unless otherwise cited), the 50% inhibitory concentration (IC_50_) in liver cell lines reported in the ChEMBL database (unless otherwise cited), and the potential factors that influence bioavailability derived from DrugBank (unless otherwise cited).

Compound	IC_50_ in Human Liver Cell Lines—ChEMBL Database [88]	Blood Levels—Human Metabolome Database [87]	Bioavailability Influencing Factors—DrugBank
Butyrate	6.17–10 mM [127,128] or 39 mM in butyric acid formulation using inhibitory protein concentration [129]	1.0 (0.3–1.5) µM [87]	Primary energy source for gut colonocytes up to 70–90%. Little to no release in peripheral system by endogenous production as most is metabolized in liver and used for gluconeogenesis [130,131].
Acetate	57 mM in acetic acid formulation using inhibitory protein concentration [129]	26.8–69.14 µM [87]	Endogenously released into peripheral blood [130,131] and used as substrate for lipogenesis, cholesterol synthesis and uptake in other organs (adipose, skeletal muscle) [132].
Propionate	45 mM in acetic acid formulation using inhibitory protein concentration [129]	0.9 ± 1.2 µM [87]	Little to no release in the peripheral system by endogenous production. Most metabolized in the liver and used for gluconeogenesis [130,131].
Resveratrol	0.05–0.354 mM [88]	N/A in blood [133] 0.006–0.028 µmol/mmol creatine in urine [87]	High absorption in gut but very low bioavailability, with rapid hepatic metabolization and excretion. Concentrations as low as 4 μM in plasma may suffice to observe pharmacological effects [134].
Curcumin	0.016–50 mM [46,88]	0.17 ± 0.013 µM [87]	Rapid intestinal metabolism and intensive second metabolism in the liver. Well-tolerated. Dose-limiting toxicity not observed [135].
Berberine	0.00 pro20–435 mM [88,121]	0.0013 ± 0.0012 µM [87]	Poor oral absorption and low bioavailability [136] due to extensive intestinal first-pass elimination [137]. Gut microbiome influences intestinal absorption and metabolic products [138].
Chlorogenic acid	0.306196 mM [58]	0.040 (0.010–0.030) µM [87]	One third directly absorbed by the intestinal tract, where up to 70% is metabolized. Shows appropriate safety profile in humans, with no apparent adverse effects [139].
Vitamin E	unknown	21.3 (12.0–80.8) µM [87]	Process of vitamin E elimination is strict and sufficiently self-regulating that vitamin E toxicity is exceedingly rare [135].
